# Genetic analysis of the response to eleven *Colletotrichum lindemuthianum* races in a RIL population of common bean (*Phaseolus vulgaris* L.)

**DOI:** 10.1186/1471-2229-14-115

**Published:** 2014-04-30

**Authors:** Ana Campa, Cristina Rodríguez-Suárez, Ramón Giraldez, Juan José Ferreira

**Affiliations:** 1 Área de Cultivos Hortofrutícolas y Forestales, SERIDA, Apdo. 13, 33300 Villaviciosa, Asturias, Spain; 2 Instituto de Agricultura Sostenible, CSIC, Apdo. 4084, E-14080 Córdoba, Spain; 3 Department of Functional Biology, University of Oviedo, 33006 Oviedo, Spain

**Keywords:** Common bean, *Phaseolus vulgaris*, *Colletotrichum lindemuthianum*, Anthracnose resistance inheritance, Genetic analyses, Genetic linkage map

## Abstract

**Background:**

Bean anthracnose is caused by the fungus *Colletotrichum lindemuthianum* (Sacc. & Magnus) Lams.- Scrib*.* Resistance to *C. lindemuthianum* in common bean (*Phaseolus vulgaris* L.) generally follows a qualitative mode of inheritance. The pathogen shows extensive pathogenic variation and up to 20 anthracnose resistance loci (named *Co-*), conferring resistance to specific races, have been described. Anthracnose resistance has generally been investigated by analyzing a limited number of isolates or races in segregating populations. In this work, we analyzed the response against eleven *C. lindemuthianum* races in a recombinant inbred line (RIL) common bean population derived from the cross Xana × Cornell 49242 in which a saturated linkage map was previously developed.

**Results:**

A systematic genetic analysis was carried out to dissect the complex resistance segregations observed, which included contingency analyses, subpopulations and genetic mapping. Twenty two resistance genes were identified, some with a complementary mode of action. The Cornell 49242 genotype carries a complex cluster of resistance genes at the end of linkage group (LG) Pv11 corresponding to the previously described anthracnose resistance cluster Co-2. In this position, specific resistance genes to races 3, 6, 7, 19, 38, 39, 65, 357, 449 and 453 were identified, with one of them showing a complementary mode of action. In addition, Cornell 49242 had an independent gene on LG Pv09 showing a complementary mode of action for resistance to race 453. Resistance genes in genotype Xana were located on three regions involving LGs Pv01, Pv02 and Pv04. All resistance genes identified in Xana showed a complementary mode of action, except for two controlling resistance to races 65 and 73 located on LG Pv01, in the position of the previously described anthracnose resistance cluster Co-1.

**Conclusions:**

Results shown herein reveal a complex and specific interaction between bean and fungus genotypes leading to anthracnose resistance. Organization of specific resistance genes in clusters including resistance genes with different modes of action (dominant and complementary genes) was also confirmed. Finally, new locations for anthracnose resistance genes were identified in LG Pv09.

## Background

Plants can recognize potential pathogens via two perception systems. One of them, named pathogen- or microbe-associated molecular patterns (PAMPs or MAMPs, respectively), detects conserved molecules associated with groups of pathogens through pattern recognition receptors leading to PAMP-triggered immunity. The other evolved to recognize pathogen virulence effectors, usually through intracellular resistance proteins (R proteins), causing effector-triggered immunity (ETI). ETI corresponds to what is classically referred to as gene-for-gene, vertical or race-specific resistance [1-3]. One of the first examples of race-specific resistance in plants was described by Barrus [4,5] in the interaction of *Colletotrichum lindemuthianum* and common bean (*Phaseolus vulgaris* L.).

Anthracnose, caused by the ascomycete fungus *C. lindemuthianum* (Sacc. & Magnus) Lams.- Scrib*.*, can result in a devastating disease in common bean. Bean anthracnose has a worldwide distribution but it is particularly problematic in temperate regions. The pathogen can attack all aerial parts of the bean plant and produces round black shrunken lesions containing flesh colored spores on leaves, stem, pods and seeds. The attack of this fungus can result in premature defoliation, premature fall of flowers and pods, seed deterioration and, in extreme cases, plant death. Infected seeds are the major means of dispersal of the pathogen [6]. The pathogen shows extensive pathogenic variation, with at least 100 pathogenic variants or races reported among isolates of *C. lindemuthianum* collected worldwide [7-11] using a set of 12 differential cultivars and a standardized method to name the races [12].

Resistance to *C. lindemuthianum* generally follows a qualitative mode of inheritance where resistant and susceptible reactions are clearly differentiated. Identification of anthracnose resistance genes by classical genetics is based on the interpretation of results obtained from F_2_ segregating populations derived from two types of crosses: R × S or R × R (R is resistant and S is susceptible). Results observed in R × S crosses are used to infer the number and mode of action of genes conferring resistance to *C. lindemuthianum*, while those for R × R crosses are used to identify the specific genes involved in the reaction against this pathogen (allelism tests). Since the first anthracnose resistance gene was reported [13], many genetic analyses have been conducted to study anthracnose resistance inheritance in different bean genotypes. Up to 20 anthracnose resistance loci conferring resistance to specific races (designated as *Co-* followed by a number or a letter) have been described in common bean: *Co-1* to *Co-7, co-8, Co-9* to *Co-14* and *Co-u* to *Co-z*[14]. All anthracnose resistance genes described, except for *co-8*, exhibit complete dominance where the dominant allele conditions the resistance reaction. A complementary mode of action between two independent resistance genes has also been described using F_2_ or F_2:3_ segregating populations, being necessary the presence of both dominant alleles for expression of resistance [15-17]. Many genetic analyses assumed that the resistance to different races in a bean genotype is conferred by the same gene. Based on this hypothesis, most classical studies considered that different resistance spectra in genotypes were due to different alleles of the same gene. So, different alleles were described for genes *Co-1*, *Co-3* and *Co-4*[14].

Most identified anthracnose resistance genes were located in the genetic map of common bean: genes *Co-1, Co-x* and *Co-w* were mapped on linkage group (LG) Pv01 [18,19]; *Co-u* was located on Pv02 [18]; *Co-13* on Pv03 [20]; *Co-2* on Pv11 [21]; *Co-3*, *Co-9*, *Co-y*, *Co-z* and *Co-10* on Pv04 [19,22-26]; *Co-4* on Pv08 [19,27]; and *Co-5*, *Co-6* and *Co-v* on Pv07 [28,29]. Mapping of genes conferring resistance to several specific races revealed that some of these *Co-* genes were organized in clusters of race-specific resistance genes. Tight linkage associations of many *Co-* resistance genes have been well established on LGs Pv04, Pv07 and Pv11 [15,19,26,28] corresponding to the named clusters Co-3, Co-5 and Co-2, respectively. At a molecular level, the majority of plant R genes cloned so far encode proteins with two specific domains: nucleotide-binding-sites (NBS) and a leucine-rich repeat (LRR) domain. Genes encoding R proteins were found in tandem on chromosome regions corresponding to the Co-3 and Co-2 clusters observed in genetic analyses [30-33].

The anthracnose resistance system in common bean has been classically investigated by analyzing a limited number of isolates or races in different segregating populations. In the present study, the response against 11 *C. lindemuthianum* races was analyzed in a recombinant inbred line (RIL) population derived from the cross Xana × Cornell 49242 in which a saturated linkage map was previously developed [34,35]. The present study aims to add understanding concerning the organization and interaction between anthracnose resistance genes, and reveals a complex *P. vulgaris*–*C. lindemuthianum* interaction.

## Results

### Segregations for the eleven *C. lindemuthianum* races

Parental line Xana was susceptible to races 6, 38, 39, 357 and 453, and resistant to the remaining six races (3, 7, 19, 65, 73 and 449). Parental line Cornell 49242 was susceptible to race 73 and resistant to the remaining ten races. Table 1 shows the observed segregations for resistance to each race in the XC RIL population (see Additional file 1 for more detail of the segregation ratios expected). Segregations for resistance to races 6, 38, 39, 357, 73 and 453 conformed with the 1:1 resistant:susceptible (R:S) ratio, expected for one resistance gene or for three independent resistance genes, complementary two-by-two. Segregation for resistance to race 65 conformed with the 3:1 R:S ratio, expected for two independent genes. Segregations for resistance to races 3, 7, 19 and 449 conformed with the 5:3 R:S ratio, expected when three independent genes, two with a complementary mode of action, are involved in the resistance response.

**Table 1:** **Observed segregations for resistance to eleven *Colletotrichum lindemuthianum *** **races in the XC RIL population**

	**Parental phenotype**		**XC RIL population**				
		**Cornell**	**Observed**		**Segregation ratio**		
**Race**	**Xana**	**49242**	**R**	**S**	**Tested (R: S)**	**χ**^**2**^	***p***
6	S	R	47	38	1: 1	0.95	0.33
38	S	R	48	42	1: 1	0.40	0.53
39	S	R	40	39	1: 1	0.01	0.91
357	S	R	37	42	1: 1	0.32	0.57
73	R	S	37	40	1: 1	0.12	0.73
65	R	R	82	20	3: 1	1.58	0.21
3	R	R	67	35	5: 3	0.44	0.51
7	R	R	65	35	5: 3	0.27	0.61
19	R	R	71	35	5: 3	0.91	0.34
449	R	R	64	39	5: 3	0.01	0.94
453	S	R	50	47	1: 1	0.09	0.76

The segregation ratio considered and the chi-square goodness-of-fit test is indicated in each case. R, resistant; S, susceptible.

### Genetic analyses of resistance to races 6, 38, 39 and 357

Segregation for resistance to races 6, 38, 39 and 357 [Xana (S) × Cornell 49242 (R)] conformed with the 1:1 R:S ratio (*p* = 0.33, *p* = 0.53, *p* = 0.91, *p* = 0.57; Table 1). Contingency chi-square tests corresponding to the joint segregations for each of the resistances with markers tagging the six main anthracnose resistance clusters deviated significantly from what was expected by random in the case of markers SQ4 and SCAreoli, that tagged the Co-2 resistance cluster on LG Pv11 (Table 2).

**Table 2:** Results of contingency chi-square tests conducted between the joint segregations for each one of the resistance to eleven races and eleven loci tagging the main anthracnose resistance clusters identified in common bean

			**Race6**	**Race38**	**Race39**	**Race357**	**Race73**	**Race65**	**Race3**	**Race7**	**Race19**	**Race449**	**Race453**
**Marker**	**LG**	**Cluster**	**χ**^**2**^**-*p***	**χ**^**2**^**-*p***	**χ**^**2**^**-*p***	**χ**^**2**^**-*p***	**χ**^**2**^**-*p***	**χ**^**2**^**-*p***	**χ**^**2**^**-*p***	**χ**^**2**^**-*p***	**χ**^**2**^**-*p***	**χ**^**2**^**-*p***	**χ**^**2**^**-*p***
CV542014	Pv01	Co-1	0.10 ns	1.76 ns	1.96 ns	3.15 ns	44.62*	12.87*	1.61 ns	2.30 ns	2.20 ns	3.16 ns	2.03 ns
OF10_530_	Pv01	Co-1	0.07 ns	0.54 ns	0.38 ns	0.88 ns	47.69*	14.95*	0.48 ns	1.40 ns	0.60 ns	1.36 ns	1.25 ns
*I*-gene	Pv02	Co-u	1.22 ns	0.62 ns	1.58 ns	0.90 ns	0.04 ns	0.01 ns	0.49 ns	0.23 ns	0.04 ns	0.07 ns	0.51 ns
SW13	Pv02	Co-u	3.57 ns	1.68 ns	2.84 ns	0.66 ns	0.11 ns	0.12 ns	0.04 ns	0.19 ns	0.42 ns	0.07 ns	0.00 ns
254-G15F_550_	Pv04	Co-3	0.32 ns	0.00 ns	0.06 ns	0.06 ns	0.65 ns	0.20 ns	15.62*	13.11*	14.11	11.85 ns	17.02*
SW12	Pv04	Co-3	0.41 ns	0.05 ns	0.31 ns	0.63 ns	0.21 ns	1.83 ns	17.99*	19.11*	16.58	13.91*	26.24*
*Pha*	Pv07	Co-5	0.10 ns	0.00 ns	0.59 ns	0.17 ns	1.19 ns	0.72 ns	0.17 ns	0.00 ns	0.09 ns	0.10 ns	0.31 ns
SZ4b	Pv07	Co-5	0.01 ns	0.17 ns	0.06 ns	0.10 ns	2.06 ns	1.11 ns	0.03 ns	0.05 ns	0.08 ns	0.00 ns	3.14 ns
SBB14	Pv08	Co-4	0.00 ns	0.04 ns	0.05 ns	0.13 ns	1.47 ns	1.11 ns	0.12 ns	0.07 ns	0.35 ns	0.02 ns	0.03 ns
SQ4	Pv11	Co-2	52.60*	57.96*	45.06*	51.19*	2.11 ns	11.43 ns	32.54*	32.75*	38.09*	35.56*	12.45*
SCAreoli	Pv11	Co-2	56.44*	62.82*	51.11*	57.74*	2.31 ns	13.33	35.24*	39.13*	41.90*	38.89*	14.54*

LG = linkage group; ns = not significant, * = significant considering a Bonferroni-correted significance threshold level of 0.0005 for multiple comparisons.

**Figure 1:**
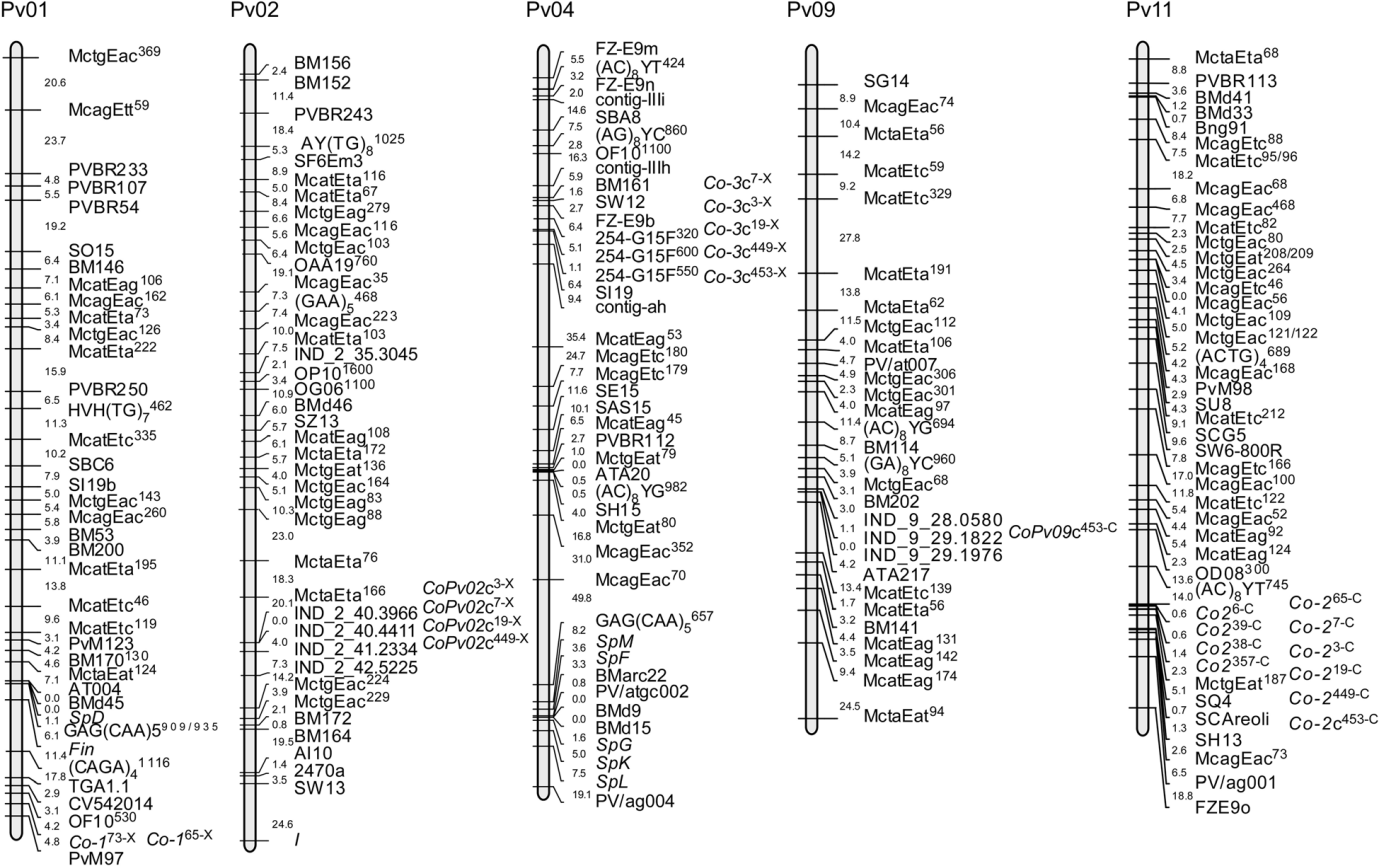
**Bean linkage groups in which anthracnose resistance genes were directly or indirectly located.** Five race-specific resistance genes were mapped on LGs Pv01, in a position corresponding to that of Co-1 resistance cluster (gene *Co1*^*73-X*^), and Pv11, in a position corresponding to that of Co-2 cluster (genes *Co-2*^*6-C*^*, Co-2*^*39-C*^*, Co-2*^*38-C*^*, and Co-2*^*357-C*^). Ten resistance genes were located on LGs Pv02 (*CoPv02c*^*3-X*^*, CoPv02c*^*7-X*^*, CoPv02c*^*19-X*^*CoPv0c2*^*449-X*^), Pv04, in a position corresponding to that of Co-3 cluster (*Co-3c*^*3-X*^*, Co-3c*^*7-X*^*, Co-3c*^*19-X*^, *Co-3c*^*449-X*^, and *Co3c*^*453-X*^), and Pv09 (*CoPv09c*^*453-C*^). Resistance genes are named by using its location in the genetic map (LG or Co-anthracnose resistance cluster), name of the isolate or race (in superscript), followed by the bean genotype in which the resistance gene was identified (X, Xana; C, Cornell 49242). Genes with a complementary mode of action are indicated using the letter ‘c’ after the name of the gene.. Map distances, on the left, are expressed in centiMorgans, estimated using the Kosambi mapping function. M_ , AFLP marker loci; Sp, seed protein marker loci; conting, FZ_, BM_ and PV, microsatellite marker loci; S_ and 254_, SCAR marker loci; *Fin*, locus controlling indeterminate versus determinate growth habit; *I*, gene controlling resistance against bean common mosaic virus; O_. RAPD markers; IND_, InDel markers.

To determine if a single gene or different genes of the same cluster were involved in the response, co-segregations for resistance to races 6, 38, 39 and 357 were considered. Co-segregation for resistance to the four races was observed in 69 RILs (35 RILs were resistant to the four races, R^6^ R^38^ R^39^ R^357^; and 34 were susceptible, S^6^ S^38^ S^39^ S^357^), and in three cases evidence of recombination was found (one RIL with each of the following haplotypes: R^6^ S^38^ R^39^ S^357^, R^6^ R^38^ R^39^ S^357^ and R^6^ S^38^ S^39^ S^357^). Accordingly, it can be concluded that four different closely linked genes at the Co-2 resistance cluster in Cornell 49242 determined specific resistance to races 6, 38, 39 and 357 (genes *Co-2*^6-C^, *Co-2*^38-C^, *Co-2*^39-C^ and *Co-2*^357-C^). Since all evidence suggests a monogenic control of resistance to each of the four races, these resistance genes were directly included in the genetic map (Figure 1).

### Genetic analyses of resistance to race 73

Resistance to race 73 [cross Xana (R) × Cornell 49242 (S)] conformed with the 1:1 R:S segregation ratio (*p* = 0.73; Table 1). The contingency chi-square values (Table 2) deviated significantly from the expectation of random segregation when compared with markers CV542014 and OF10_530_, tagging the Co-1 resistance cluster. This result indicates that resistance to race 73 in Xana may be mediated by one resistance gene (*Co-1*^73-X^) located at the Co-1 cluster. Genetic mapping confirmed the position of this gene at the end of LG Pv01, in a position corresponding to cluster Co-1 (Figure 1).

### Genetic analyses of resistance to race 65

Segregation of resistance to race 65 [cross Xana (R) × Cornell 49242 (R)] suggests the presence of two independent dominant genes (Table 1). The chi-square values deviated significantly from the expectation of random segregation when resistance to race 65 was compared with molecular markers that tagged the Co-1 and Co-2 regions (Table 2). This result suggests the localization of one resistance gene at the Co-1 cluster, and a second gene at the Co-2 cluster. To confirm this model, segregation for resistance to race 65 was analyzed in six subpopulations established from the total XC RIL population (see Additional file 2): X-Co-1 and C-Co-1, formed by 30 and 47 RILs showing, respectively, the Xana and Cornell49242 alleles for the markers CV542014 and OF10_530_. X-Co-2 and C-Co-2, formed by 42 and 41 RILs showing, respectively, the Xana and Cornell49242 alleles for the markers SQ4 and SCAReoli. X-Co-3 and C-Co-3, formed by 37 and 41 lines, respectively, the Xana and Cornell49242 alleles for markers 254-G15F_550_ and SW12. In the X-Co2 subpopulation, the resistance to race 65 segregated according to a 1:1 R:S ratio, expected in the case of one resistance gene. A linkage analysis carried out within this subpopulation revealed a close linkage between this segregating gene and *Co-1*^73-X^ (RF = 0.00; LOD = 10.54). The localization of a resistance gene for race 65 derived from Xana at the Co-1 cluster was confirmed in the X-Co1 subpopulation, in which the Co-1 region from Xana was fixed; the 27 RILs of this subpopulation were resistant to race 65. The opposite subpopulation, C-Co1, showed a segregation for resistance to race 65 according to the ratio expected for one dominant gene (i.e. 1:1 R:S). This resistance gene exhibited a close linkage with *Co-2*^38-C^ (RF = 0.04; LOD = 7.68). The localization of a resistance gene against race 65 derived from Cornell 49242 at the Co-2 cluster was confirmed in the C-Co2 subpopulation, in which the majority of lines showed a resistant phenotype against race 65 (all except for two lines, probably due to recombination). Independence between resistance against race 65 and the Co-3 chromosome region was deduced from subpopulations X-Co3 and C-Co3 – in both these subpopulations there was a 3:1 R:S ratio for resistance to race 65, as for the total XC population.

It can be concluded that resistance to race 65 was conferred by one gene (*Co-1*^65-X^) in Xana located at the Co-1 cluster on Pv01 and by a gene (*Co-2*^65-C^) in Cornell 49242 located at the Co-2 cluster on Pv11 (see Figure 1).

### Genetic analyses of resistance to races 3, 7, 19 and 449

Segregation of resistance to races 3, 7, 19 and 449 [Xana (R) × Cornell (R)] conformed with the 5:3 R:S ratio (*p* = 0.51, *p* = 0.61, *p* = 0.34, *p* = 0.94 respectively; Table 1) expected for three independent genes, two of them having a complementary mode of action. Resistance to four races showed close co-segregation. A total of 96 lines showed the parental genotypes (63, R^3^ R^7^ R^19^ R^449^ and 33, S^3^S^7^S^19^S^449^), while only four lines revealing evidences of recombination (2, R^3^ S^7^ R^19^ R^449^; and 2, R^3^ R^7^ R^19^ S^449^).

Concerning resistance to race 3, the contingency chi-square values deviated significantly from the expectation of random segregation when resistance to this race was compared with molecular markers tagging the Co-3 and Co-2 chromosome regions (Table 2). This result can be interpreted as one of the resistance genes being located at the Co-3 cluster and a second gene located at the Co-2 cluster. Concerning the third complementary gene, it segregated independently from the molecular markers tagging the Co-1, Co-5, Co-4 and Co-u regions (Table 2), so contingency chi-square tests were conducted between resistance to race 3 and the remaining 368 loci included in the XC genetic map. There was significant deviation, with a block of three markers closely mapped on LG Pv02 (Figure 1): IND_2_403966 (Cont. χ^2^ = 4.24, p = 0.04); IND_2_404411 (Cont. χ^2^ = 4.51, p = 0.03) and MctaEta^166^ (Cont. χ^2^ = 5.24, p = 0.02).

Analyses within subpopulations (see Additional file 2) were consistent with this scenario. Within subpopulations involving the Co-1 cluster (X-Co1 and C-Co1) resistance to race 3 fitted a 5:3 R:S ratio, as for the total XC RIL population, suggesting that the Co-1 region was not involved in genetic control against race 3. However, a change in the segregation ratio was observed within the two subpopulations involving the Co-3 cluster. Good fits to 3:1 and 1:1 R:S ratios were observed in the X-Co-3 and C-Co-3 subpopulations, respectively. This is the expected situation if one complementary gene proceeding from Xana is located at the Co-3 cluster. Concerning the Co-2 cluster, most lines included in the C-Co-2 subpopulation were resistant to race 3 (except one susceptible line, probably due to recombination); and the opposite X-Co2 subpopulation fitted a 1:3 R:S ratio, expected for two independent and complementary genes (see Additional file 1 for more detail). Both results are expected if the resistance gene derived from Cornell 49242 without a complementary mode of action was located at the Co-2 cluster. Two subpopulations involving LG Pv02 were also considered for race 3 using markers IND_2_403966 and MctaEta^166^: the X-Pv02 subpopulation including 26 RILs showing the Xana allele for both markers, and the C-Pv02 subpopulation including 24 RILs showing the Cornell 49242 alleles. In the X-Pv02 subpopulation resistance to race 3 fitted a 3:1 R:S ratio (21:5 R:S; χ^2^ = 0.46, p = 0.50), while the C-Pv02 subpopulation fitted a 1:1 R:S ratio (12:12 R:S; χ^2^ = 0.00, p = 1.00). This situation supports the localization of a complementary resistance gene from Xana against race 3 in this position of LG Pv02.

In summary, results indicate involvement of three resistance genes against race 3 (Figure 1): one gene (*Co-2*^3-C^) from Cornell 49242 located at the Co-2 cluster, and two independent resistance genes from Xana with a complementary mode of action, one (*Co-3*c^3-X^) located at the Co-3 cluster on Pv04 and a second (*CoPv02*c^3-X^) on LG Pv02. The same model deduced for resistance to race 3 is valid for resistance to races 7, 19 and 449, all genes showing close co-segregation.

### Genetic analyses of resistance to race 453

Resistance to race 453 [cross Xana (S) × Cornell 49242 (R)] fitted a 1:1 R:S segregation ratio (*p* = 0.76; Table 1). In this case, the chi-square values (Table 2) deviated significantly from the expectation of random segregation when compared with molecular markers that tagged two different chromosome regions: SW12 and OF10_1100_ (Co-3 region) and SQ4 and SCAreoli (Co-2 region). This finding provides evidence concerning the presence of more than one gene in the resistance response against race 453. Given the observed ratio, the most probable explanation is that resistance to race 453 was controlled by three independent resistance genes having a complementary mode of action two-by-two (see Additional file 1 for more detail). According to the results (Table 2), one of these genes would be located at the Co-2 region and another probably located at the Co-3 region. The third complementary gene segregated independently from molecular markers tagging the Co-1, Co-5, Co-4 and Co-u regions, so contingency chi-square tests between the resistance to race 453 and the remaining 368 loci included in the XC genetic map were conducted. A significant deviation was observed with a block of 11 markers mapped on LG Pv09, from marker BM202 to McatEag^174^ (see Figure 1 and Table 3).

**Table 3:** Genotype in the parental Xana and the recombinant inbred lines XC30, and XC88 for a block of markers in LGs Pv04, Pv09 and Pv11 of lines selected in order to verify the location and mode of action of the resistance genes deduced against race 453

**Region or cluster**				**Race 453**		**Genotype**^**a**^		
	**LG**	**Marker loci**	**cM**	**Cont.χ**^**2**^	***p***	**Xana**	**XC30**	**XC88**
Co3	Pv04	SW12	-	26.24	*	A	B	B
	Pv04	FZ-E9b	2.7	18.36	*	A	A^b^	B
	Pv04	254-G15F^320^	6.4	8.52	*	A	B	A^b^
	Pv04	254-G15F^600^	5.1	16.62	*	A	B	B
	Pv04	254-G15F^550^	1.41	17.02	*	A	B	B
CoPv09	Pv09	IND_9_280580	-	4.50	*	A	A	B
	Pv09	IND_9_291822	1.1	8.51	*	A	A	B
	Pv09	IND_9_291976	0	8.17	*	A	A	B
Co2	Pv11	*Co-2*^6-C^	-	14.43	*	A	B	A
	Pv11	*Co-2*^39-C^	0.6	15.62	*	A	B	A
	Pv11	*Co-2*^38-C^	0.6	18.27	*	A	B	A
	Pv11	*Co-2*^357-C^	1.4	19.49	*	A	B	A
	Pv11	MctgEat^187^	2.3	16.44	*	A	B	A
	Pv11	SQ4	5.1	12.45	*	A	B	A
	Pv11	SCAreoli	0.7	14.54	*	A	B	A

LG = linkage group. cM, distance between loci in centimorgans.

* = significant.

^a^A = genotype of the parental line Xana; B = genotype of the parental line Cornell49242.

^b^recombinant genotypes.

To confirm this model, segregation for resistance to race 453 was analyzed within the subpopulations established (Additional file 2). Segregation for resistance in the X-Co1 and C-Co1 subpopulations fitted a 1:1 R:S ratio, as for the total RIL population, suggesting that this region was not involved in genetic control of the reaction to race 453. In contrast, a change in the segregation ratio was observed in the remaining subpopulations. In the X-Co2 and C-Co-3 subpopulations resistance to race 453 fitted a 1:3 R:S ratio, expected in the case of two complementary and independent genes. This result suggests that both subpopulations lacked one of the three complementary resistance genes. The X-Co2 subpopulation lacked a complementary resistance gene from Cornell 49242 located on the Co-2 region while the C-Co3 subpopulation lacked a complementary resistance gene from Xana, located at the Co-3 region. The observed segregation within the opposite subpopulations, C-Co2 and X-Co3, conformed with the 3:1 R:S ratio, thus being consistent with this scenario. If one of the three complementary genes was fixed in a subpopulation, then a 3:1 R:S ratio would be expected.

For the third resistance gene against race 453, with location estimated at LG Pv09 based on contingency chi-square tests (Table 3), two additional subpopulations were considered using markers IND_9_280580 and ATA217. In the X-Pv09 subpopulation, formed by 46 RILs showing the Xana allele for both markers, resistance to race 453 fitted a 3:1 R:S ratio (16:9 R:S, χ^2^_3:1_ = 1.61, *p* = 0.20). In the C-Pv09 subpopulation, formed by 30 RILs showing the Cornell 49242 allele, resistance to race 453 fitted a 1:3 R:S ratio (15:24 R:S, χ^2^_1:3_ = 3.77, *p* = 0.05). This result is in agreement with the localization of a third complementary resistance gene from Cornell 49242 at LG Pv09.

In summary, results indicate the involvement of three complementary genes, complementary two-by-two in the genetic control of response to race 453 (Figure 1): one gene (*Co3*c^453-X^) from Xana located on the Co-3 region, one (*Co2*c^453-C^) from Cornell 49242 located on the Co-2 region and a third gene (*CoPv09c*^453-C^) from Cornell 49242 located on LG Pv09.

### Genetic dissection of resistance to race 453

Genetic dissection was performed to verify the complex system of resistance observed against race 453. Three susceptible lines were selected according to their parental genotype (Xana or Cornell 49242) for markers located in LGs Pv04, Pv09 and Pv11 (Table 3), in which were tentatively localized the three complementary resistance genes. Parental line Xana was susceptible to race 453 and according to this model it carried the complementary gene *Co-3*c^453-X^ and lacked *Co-2*c^453-C^ and Co*Pv09c*^*453-C*^. RIL XC30 showed the Cornell 49242 genotype for markers of the Co-3 cluster, and the Xana genotype for markers that tagged the other two regions, the Co-2 cluster and the Pv09 region. Based on this, susceptible line XC30 should have the *Co-2*c^453-C^ gene and lack *Co-3*c^453-X^ and *CoPv09*c^453-C^. Finally, RIL XC88 showed the Cornell genotype for markers tagging the Pv09 region and the Xana genotype for markers of clusters Co-2 and Co-3. Thus, it should have Co*Pv09*c^453-C^ and lack *Co-3*c^453-X^ and *Co-2*c^453-C^.

A total of 13 F_1_ seedlings were obtained from the cross between the susceptible lines Xana (*Co-3*c^453-X^) × RIL XC30 (*Co-2*c^453-C^) and all were resistant to race 453, indicating a complementary mode of inheritance. A total of 16 F_1_ seedlings were obtained from the cross Xana (*Co-3*c^453-X^) × RIL XC88 (*CoPv09c*^*453-C*^) and all were resistant to race 453. Finally, from the cross RIL XC88 (*CoPv09c*^*453-C*^) × RIL XC30 (*Co-2*c^453-C^) a total of 23 seedlings were obtained, all resistant to race 453. In all cases, cross authenticity was verified using codominant molecular markers. This result confirmed the model of three genes, complementary two-by-two, involved in the genetic control of resistance to race 453 in the XC RIL population.

## Discussion

In the present study, the inheritance of resistance to 11 *C. lindemuthianum* isolates classified as different races was analyzed in a RIL population derived from the cross Xana × Cornell 49242. In classical genetic analysis, the identification of a resistance gene is based its relationship of independence or linkage with other genes previously described, through allelism tests. Molecular markers can help in determining the identity of a resistance gene. Molecular markers linked to the main anthracnose resistance genes have been reported [14] and can be used to establish the identity of a resistance gene by means of linkage analyses. The use of saturated genetic linkage maps allows more accurate location of a resistance gene by direct mapping, and also the identification of new resistance loci, independent of those previously described. Nevertheless, the use of linkage maps for direct mapping is limited by the number of genes involved in the resistance, and/or by possible epistatic interaction between them. This scenario occurred for the anthracnose resistance genetic analyses conducted on the XC RIL population. Only reactions to races 6, 38, 39, 357 and 73 exhibited a monogenic segregation and the respective resistance genes involved were directly mapped. Resistance to the remaining six races showed complex segregations, involving several Mendelian genes with different modes of action. In this case, genetic analyses supported by contingency chi-square tests and subpopulation analyses were proposed as an alternative solution for the analyses of complex segregations involving more than one gene. Genome-wide analysis using contingency chi-square tests were conducted to identify the regions of the genetic map showing a significant association with the reaction to a specific race. Significant associations between the response to a specific race and a mapped locus suggest a genetic linkage relationship between them, particularly if significant deviations are caused by an excess of the parental classes. Subpopulation analysis allows simplification of segregation and testing the involvement of a specific region in the control of the resistance. However, subpopulation size is reduced in respect to that of the original population, so larger original populations are required. Finally, genetic dissection was performed in specific cases to check the position of a resistance gene and its mode of action. Nevertheless, genetic dissection requires the development of new segregating populations from genotypes showing a specific combination of alleles, and these genotypes are not always available within the original segregating population due to recombination events. This systematic genetic analysis has allowed the drawing of conclusions concerning genetic control of anthracnose resistance in parental lines Xana and Cornell 49242.

Results indicate that Cornell 49242 carried a complex cluster of race-specific resistance genes at the end of LG Pv11, corresponding to the Co-2 cluster. Four resistance genes (*Co-2*^6-C^, *Co-2*^38-C^, *Co-2*^39-C^ and *Co-2*^357-C^) were directly mapped on this cluster, closely linked to markers SQ4 and SCAreoli. Recombinant lines in the response to the four races were detected, indicating that different resistance genes were present. From the analyses of subpopulations, the presence of another six resistance genes at this position was indirectly deduced: *Co-2*^3-C^, *Co-2*^7-C^, *Co-2*^19-C^, *Co-2*^65-C^ and *Co-2*^449-C^, as well as *Co-2*c^453-C^ with a complementary mode of action. The dominant resistance gene *Are* (re-named *Co-2*) was reported in Cornell 49242 protecting against races alpha, beta and gamma [36]. Resistance genes showing a complementary mode of action have been also suggested in this genotype from the observed segregations in F_2_ populations [17]. Gene *Are* was introgressed into cultivar Ms8EO2 from Cornell 49242 [21,37]. Using a BC_1_F_1_ population obtained from the backcross (Ms8EO2/*Corel), gene *Are* was mapped at the end of LG P1 (corresponding to LG Pv11) closely linked to the SCAR marker SCAreoli [37]. Results of the present study indicated that the original gene *Are* described in Cornell 49242 was in fact a cluster of linked resistance genes with each one conditioning resistance to different races of *C. lindemuthianum*. A cluster organization including closely linked genes against races 6, 31, 38, 39 and 65, was also identified in the A252 bean genotype in the same genetic position using the F_2:3_ population derived from the cross Andecha × A252 [19].

Results also indicated that genotype Cornell 49242 carried two complementary resistance genes to race 453, one (*Co-2c*^*453-C*^) located in the described cluster Co-2, and another (*CoPv09c*^*453-C*^) in LG Pv09 between markers BM202 and ATA217 – this is the first anthracnose resistance gene located in this LG. Interestingly, a quantitative trail locus (QTL) involved in resistance against *Pythium ultimum*[38] was localized to this same relative position. Evidence supplied by *in silico* analysis in the sequenced bean genotype G19833 (http://www.phytozome.net) was consistent with the location of anthracnose resistance genes in this position*.* At least four genes encoding NBS-LRR proteins and four genes encoding protein kinases, all typical R proteins, were annotated between markers BM202 and ATA217 (see Additional file 3).

Genetic control of anthracnose resistance in the cultivar Xana had not been previously analyzed. Xana showed a resistant reaction against six of the eleven isolates analyzed. Genetic analysis suggested that regions on LGs Pv01, Pv02, Pv04 and Pv11 were implicated in the response. Resistance to race 73 showed monogenic inheritance and the corresponding locus (*Co-1*^73-X^) was directly mapped on the end of LG Pv01. Subpopulation analysis allowed the deduction of the presence of one additional resistance gene (*Co-1*^65-X^) in this relative position. *Co-1*^65-X^ probably corresponds to the resistance gene against race 65 mapped in this same relative position from the genotype Andecha [19], as Andecha is one of the parents from which Xana was obtained. The *Co-1*^73-X^ gene is closely linked to markers OF10_530_, TGA1.1 and CV542014. The OF10_530_ fragment was previously linked to the anthracnose resistance locus *Co-1* in the F_2:3_ population derived from the cross between the near-isogenic lines N85006 S and N85006 R [39] and later mapped to Pv01 [18,19,40]. A gene conferring resistance to race 73 in AND277 was also located on Pv01 using a F_2_ population derived from the cross AND277 × Rudá [41]. This resistance gene in AND277, that corresponds to *Co-1,* is closely linked to markers CV542014 and TGA1.1. In the genotype Kaboon, *Co-1* was also shown to have a cluster organization, including three resistance genes against races 31, 81 and 1545 [15]. In conclusion, different genetic evidence supported the presence of a cluster, including race-specific resistance genes to anthracnose in the relative position in which the *Co-1* gene was mapped (named cluster Co-1).

Resistance genes derived from Xana against races 3, 7, 19 and 449 were indirectly located on LGs Pv02 and Pv04, all with a complementary mode of action. A gene conferring specific resistance to *C. lindemuthianum* strains E4 and E42b (showing complete co-segregation) was mapped to the end of LG Pv02 in the Mesoamerican genotype BAT93 using the RIL population BAT93 × JALOEEP558. This gene, named *Co-u*, was located in the vicinity of the *I* locus [18], a complex resistance cluster effective against potyviruses [42]. The correspondence between *Co-u* and the resistance cluster identified in Xana is not clear, because the *I* locus and the SW13 marker (linked to gene *I*) are included in the XC genetic map, and segregated independently from resistance genes *CoPv02c*^*3-X*^, *CoPv02*c^7-X^, *CoPv02*c^19-X^ and *CoPv02*^449-X^. The physical position of these resistance genes was estimated to be around 40 Mbp of chromosome 2, based on the physical position of the InDel markers. *In silico* analysis (http://www.phytozome.net) was consistent with the location of genes involved in a resistance response in thisposition: eleven genes encoding NBS-LRR proteins, or protein kinases, were annotated in genotype G19833 between 39.860 and 40.926 Mbp of chromosome 2 (see Additional file 3 for more detail).

The location of five resistance genes from Xana on LG Pv04 (*Co-3*c^3-X^, *Co-3*c^7-X^, *Co-3*c1^9-X^, *Co-3*c^449-X^ and *Co-3*c^453-X^), all showing a complementary mode of action, was also deduced from the genetic analysis. Different anthracnose resistance specificities were mapped in this relative position from the bean genotypes Mexico222, Widusa, BAT93, JaloEEP558, MDRK, Kaboon, A252 and A493 [15,18,19,23,25,26,33]. In genotype Kaboon, one of the genes located at this position exhibited a complementary inheritance against race 31 [15]. On this position were mapped the well-known resistance genes *Co-3* and *Co-9*[14]. In this LG, but in a distal position from *Co-9*, was mapped *Co-10* from cultivar Ouro Negro as a single gene conferring resistance against races 23, 64, 73, 81 and 89 [22,24]. To date, independence between *Co-10* and resistance genes included in the Co-3 cluster has not been clearly established.

This study confirmed a wide variation in the fungus–plant interaction. Up to 22 race-specific genes could be involved in the resistance to 11 isolates. Alternative splicing generates multiple transcript variants from single genes and could explain the observed variation in instances in which recombination among race-specific loci was not detected. Alternative splicing was found for the *RCT1* gene in *Medicago truncatula* that confers resistance to multiple races of *C. trifolli*[43]. A complementary mode of action indicates the cooperation of two genes (or proteins) in the resistant reaction. Combination of several proteins in the recognition of the fungus can be a mechanism generating variation in the interaction; the number of possible combinations for resistance increases. In the guard and decoy models, the effector modifies an accessory protein, which may be its virulence target (guard model) or a structural mimic of such a target (decoy model). The modified accessory protein is recognized by the NBS-LRR receptor. Under the bait model, interaction of an effector with an accessory protein facilitates direct recognition by the NBS-LRR receptor [1,44]. Two resistance genes could act in a complementary way under any of the three models, with the presence of the two proteins necessary to trigger the resistance response. Complementary resistance genes required for mediated resistance pathways have been described in common bean (against the bean rust pathogen *Uromyces appendiculatus*[45]), tomato [46,47], *Arabidopsis*[48] and barley [49]. However, no references were found in a model involving three resistance genes which are complementary two-by-two. It is important to note that only if the two parents differ in the two complementary genes, this type of genetic control can be detected. Segregation corresponding to a single gene would be expected if the parental lines differ in only one of the two complementary genes. In consequence, the resistance gene detected in a bean genotype against a *C. lindemuthianum* isolate can depend on the segregating population investigated.

## Conclusions

Detailed knowledge of the genetic control of traits (in this case, resistance to anthracnose) is relevant for the identification of genomic sequences involved in the expression using a positional cloning approach, and the development of plant breeding programs. Results confirmed that the response in the *P. vulgaris*–*C. lindemuthianum* interaction was very specific, conditioned by both pathogenic variation of the fungus and the bean genotype. Genetic control of the resistant reaction in common bean can be controlled by different race-specific resistance genes depending on the bean genotype. Many of these genes were organized as clusters of closely linked race-specific genes located in specific chromosome regions, and these resistance genes can exhibit dominant epistatic interaction or complementary mode of action. The identification of resistance clusters suggest that most of the described alleles for the *Co-1* and *Co-3* loci are different and that closely linked loci control the resistance reaction against different races or isolates in a bean genotype. All these aspects should be considered in interpretation of the genetic analysis and the reported results.

## Methods

### Plant material

A population of 104 F_7_ RILs developed from the cross Xana × Cornell 49242 was used in this study [34,35]. The population (XC population) was obtained by single seed descent method from individual F_2_ plants. Xana is a bean variety developed at Servicio Regional de Investigación y Desarrollo Agroalimentario (SERIDA, Villaviciosa, Spain) originated from a cross between the landraces Andecha and V203. It is a very large white-seeded line, with determinate growth habit and belongs to the fabada market class. Cornell 49242 is a very small black-seeded line included in the black turtle market class, with indeterminate prostrate growth habit. Cornell 49242 is one of the 12 common bean anthracnose differential cultivars used to identify *C. lindemuthianum* races [12]. The remaining 11 differential cultivars were used as a control to confirm the identity of the *C. lindemuthianum* races.

### Inoculation procedure and disease scoring

Eleven isolates of *C. lindemuthianum* classified in different races according to Pastor-Corrales [12] were used in this study: races 7, 39, 65, 73, 357, 449 and 453, from the collection of the Crop and Soil Sciences Department (Michigan State University, USA); and races 3, 6, 19 and 38 from the SERIDA collection. All isolates were obtained from monosporic cultures maintained in fungus-colonized filter paper at –20°C for long-term storage. To obtain abundant sporulation, the isolates were grown at 21°C in darkness for 10 d in potato dextrose agar (DIFCO, Becton Dickinson and Company, Sparks, MD, USA). Spore suspensions were prepared by flooding the plates with 5 ml of 0.01% Tween 20 (Sigma-Aldrich, St. Louis, MO, USA) in sterile distilled water and scraping the surface of the culture with a spatula. Inoculations were carried out by spraying 8–10-d-old seedlings with a spore suspension containing 1.2 × 10^6^ spores/ml. The seedlings were maintained in a climate chamber at 20–22°C, 95–100% humidity and 12-h photoperiod. Responses of the plants were evaluated after 7–9 d using a 1–9 scale [50]. Seedlings with no visible symptoms (severity value 1) or showing limited necrotic lesions (severity values 2–3) were considered resistant (R^x^ = resistant reaction against race X). Seedlings with large sporulating lesions (severity values 4–8) or dead (severity value 9) were considered susceptible (S^x^ = susceptible reaction against race X).

The response to a specific race in the XC population was evaluated by inoculating all recombinant lines in the same test, including 8–10 seedlings per line. The parents Xana and Cornell 49242 and the remaining 11 common bean anthracnose differential cultivars were also included in each test.

Resistance genes were named by using their location in anthracnose resistance clusters (Co-cluster), name of the isolate or race (in superscript) followed by the bean genotype in which the resistance gene was identified. For example, a gene conferring resistance to race N in Cornell 49242 located in the Co-2 cluster was identified as *Co-2*^N-C^. If a resistance gene was not located in regions in which Co genes were previously mapped, it was designated using the LG in which its position was estimated. For example, a gene conferring resistance to race M in Cornell 49242, located in LG Pv09, was tentatively named *CoPv09*^*M-C*^. Genes with a complementary mode of action were indicated using the letter ‘c’ after the name of the gene (e.g*. Co-2c*^*N-C*^).

### Genetic linkage map

A genetic linkage map developed in the XC RIL population was used as a support for the localization of the chromosome regions involved in the resistance response to different anthracnose races. The map consisted of 379 loci distributed across 11 LGs [34,35]. MAPMAKER Macintosh version 2.0 software [51] was used for the map construction using a log of the likelihood ratio (LOD) threshold of 3.0 and a recombination fraction of 0.25. Marker order was estimated based on multipoint compare, order and ripple analyses. Distances between ordered loci (in centimorgans) were calculated using the Kosambi mapping function. The resulting map had 11 LGs, which were aligned according to the common bean core linkage map using common molecular markers as anchor points. LGs were named according to Pedrosa-Harand et al. [52].

The XC genetic map included markers tagging the regions in which anthracnose resistance loci were located: markers CV542014 and OF10_530_ tagging the anthracnose resistance cluster Co-1 (LG Pv01) [41]; gene *I,i* and SW13 marker for the *Co-u* resistance gene on LG Pv02 [18,53]; 254-G15F and SW12 tagging the resistance cluster Co-3 (Pv04); seed protein Phaseolin (Pha) and marker SZ4b for the resistance cluster Co-5 (Pv07); marker SBB14 for cluster Co-4 (Pv08); and markers SQ4 and SCAreoli which tag anthracnose resistance cluster Co-2 (Pv11). Recently, a new anthracnose resistance gene in common bean, identified as *Co-13,* was located on LG Pv03, linked to RAPD marker OPV20_700_[20]. This marker was not polymorphic in the XC RIL population, although its relative position on LG Pv03 should be represented across the 35 loci forming this LG in the XC linkage map.

### Genetic analyses

Goodness-of-fit of observed to expected ratios was tested by chi-square. Additional file 1 summarizes segregation ratios expected in a RIL population under different hypotheses, considering one to three genes and different modes of action.

To identify the gene(s) involved in the resistance to a specific isolate, a systematic genetic analysis was performed as follows:

i) Contingency chi-square analyses. Contingency chi-square tests of the joint segregation for each scored resistance with each marker included on the XC linkage map were performed. A significant deviation from random segregation would suggest that the chromosome region tagged with the marker could be involved in the resistance response. Significance thresholds were determined using Bonferroni correction from α-level of 0.05 [54]. First, this analysis was focused on markers tagging the main six chromosome regions which included Co genes (*Co-1* on LG Pv01; *Co-2* on LG Pv11; *Co-3* on LG Pv04, *Co-4* on LG Pv08, *Co-5* on LG Pv07 and the region of LG Pv02 in which the Co-u gene was mapped). If a resistance gene was not localized in one of the main anthracnose resistance clusters, the contingency chi-square test was conducted using the remaining 368 loci included in the XC genetic map.

ii) Direct mapping. When the segregation ratio and the contingency chi-square analysis suggested the presence of one resistance gene, it was directly included in the genetic map.

iii) Subpopulation analyses. When the segregation ratio and the contingency chi-square analyses suggested the presence of more than one resistance gene, subpopulation analyses were performed. Two subpopulations were established per region, considering the parental genotypes for the markers that tagged it. Two markers per region were used to reduce the possibility of recombination events. If a resistance gene was located in the region marked, changes in the segregation ratio compared to that of the total XC RIL population were expected in the established subpopulations. If the region considered in the subpopulations was not involved in genetic control of the resistance, no change was expected in the segregation ratio of the resistance, compared to that for the complete XC RIL population.

iv) Genetic dissection. In particular cases, relative position and mode of action of specific resistance genes were verified through crosses between selected RILs. These lines were selected considering the resistance response and their genotypes (Xana or Cornell 49242) for underlying markers located in the putative position of the genetic map in which the candidate gene was located.

## Competing interests

The authors declare that there are no competing interests.

## Authors’ contributions

AC (conducted part of the experiments, analyzed data, wrote the manuscript); CRS (conducted part of the experiments, analyzed data, revised the manuscript); RG (designed the experiments, analyzed data, revised the manuscript); JJF (designed the experiments, analyzed data, wrote the manuscript). All authors read and approved the final manuscript.

## Supplementary Material

Additional file 1 **Segregation ratios expected in a RIL population under different hypothesis.** R, resistant; S, susceptible. Click here for file

Additional file 2 Observed segregations for resistance to races 65, 3, 7, 19, 449, and 453 in six subpopulations formed from the XC RIL population for three chromosome regions, Co-1, Co-2 and Co-3. Click here for file

Additional file 3 **Candidate resistance genes annotated from 31 to 41 Mbp of chromosome 2 and from 26 to 30 Mbp of chromosome 9, respectively, using the G19833 genotype sequence available at **http://www.phytozome.net. Click here for file
