# Supplementary material for: Genetic analysis of the response to eleven Colletotrichum lindemuthianum races in a RIL population of common bean (Phaseolus vulgaris L.)
Source: BMC Plant Biol. 2014 Apr 30;14:115. doi: 10.1186/1471-2229-14-115 (PMC4021056; doi:10.1186/1471-2229-14-115)
Supplement: Additional file 1 — Segregation ratios expected in a RIL population under different hypothesis. R, resistant; S, susceptible. [file 1471-2229-14-115-S1.doc]

**Additional file 1. Segregation ratios expected in a RIL population under different hypothesis. The number of genes involved in the resistance response and their mode of action are considered. R, resistant; S, susceptible**

| Number and mode of action of the resistance genes | Genotypes in a RIL population | Reaction | Expected ratio |
| --- | --- | --- | --- |
| One gene (A_1_,A_2_) | A_1_A_1_ | Resistant | 1 R: 1 S |
|  | A_2_A_2_ | Susceptible |  |
| Two independent genes | A_1_A_1_B_1_B_1_ | Resistant | 3 R: 1 S |
| (A_1_,A_2_; B_1_,B_2_) | A_1_A_1_B_2_B_2_ | Resistant |  |
|  | A_2_A_2_B_1_B_1_ | Resistant |  |
|  | A_2_A_2_B_2_B_2_ | Susceptible |  |
| Two independent and | B_1_B_1_C_1_C_1_ | Resistant | 1 R: 3 S |
| complementary genes | B_1_B_1_C_2_C_2_ | Susceptible |  |
| (B_1_,B_2_; C_1_,C_2_) | B_2_B_2_ C_1_C_1_ | Susceptible |  |
|  | B_2_B_2_C_2_C_2_ | Susceptible |  |
| Three independent genes, two of | A_1_A_1_B_1_B_1_C_1_C_1_ | Resistant | 5 R: 3 S |
| them with a complementary | A_1_A_1_B_1_B_1_C_2_C_2_ | Resistant |  |
| mode of action | A_1_A_1_B_2_B_2_C_1_C_1_ | Resistant |  |
| (A_1_,A_2_; B_1_,B_2_; C_1_,C_2_) | A_1_A_1_B_2_B_2_C_2_C_2_ | Resistant |  |
|  | A_2_A_2_B_1_B_1_C_1_C_1_ | Resistant |  |
|  | A_2_A_2_B_1_B_1_C_2_C_2_ | Susceptible |  |
|  | A_2_A_2_B_2_B_2_C_1_C_1_ | Susceptible |  |
|  | A_2_A_2_B_2_B_2_C_2_C_2_ | Susceptible |  |
| Three independent genes, | B_1_B_1_C_1_C_1_D_1_D_1_ | Resistant | 1 R: 1 S |
| complementary | B_1_B_1_C_1_C_1_D_2_D_2_ | Resistant |  |
| two to two | B_1_B_1_C_2_C_2_D_1_D_1_ | Resistant |  |
| (B_1_,B_2_; C_1_,C_2_; D_1_,D_2_) | B_1_B_1_C_2_C_2_D_2_D_2_ | Susceptible |  |
|  | B_2_B_2_C_1_C_1_D_1_D_1_ | Resistant |  |
|  | B_2_B_2_C_1_C_1_D_2_D_2_ | Susceptible |  |
|  | B_2_B_2_C_2_C_2_D_1_D_1_ | Susceptible |  |
|  | B_2_B_2_C_2_C_2_D_2_D_2_ | Susceptible |  |
