# Supplementary material for: Genetic analysis of the response to eleven Colletotrichum lindemuthianum races in a RIL population of common bean (Phaseolus vulgaris L.)
Source: BMC Plant Biol. 2014 Apr 30;14:115. doi: 10.1186/1471-2229-14-115 (PMC4021056; doi:10.1186/1471-2229-14-115)
Supplement: Additional file 2 — Observed segregations for resistance to races 65, 3, 7, 19, 449, and 453 in six subpopulations formed from the XC RIL population for three chromosome regions, Co-1, Co-2 and Co-3. [file 1471-2229-14-115-S2.doc]

**Additional file 2. Subpopulation analyses**. Observed segregations for resistance to races 65, 3, 7, 19, 449, and 453 in six subpopulations formed from the XC RIL population for three chromosome regions, Co-1, Co-2 and Co-3.

|  |  |  | Subpopulations selected within the XC RIL population^a^ | | | | | | | | | | | | | | | | | | | | | | | | | | | | | | |
| --- | --- | --- | --- | --- | --- | --- | --- | --- | --- | --- | --- | --- | --- | --- | --- | --- | --- | --- | --- | --- | --- | --- | --- | --- | --- | --- | --- | --- | --- | --- | --- | --- | --- |
|  |  | X-Co-1 | | | | |  | C-Co-1 | | | |  | X-Co-3 | | | | | |  | C-Co-3 | | | |  | X-Co-2 | | | |  | C-Co-2 | | | |
| Race | Ratio | Ob  freq  (R:S) | | Ratio | χ^2^ | *p* |  | Ob  freq  (R:S) | Ratio | χ^2^ | *p* |  | | - | Ob  freq  (R:S) | Ratio | χ^2^ | *p* |  | Ob  freq  (R:S) | Ratio | χ^2^ | *p* |  | Ob  freq  (R:S) | Ratio | χ^2^ | *p* |  | Ob  freq  (R:S) | Ratio | χ^2^ | *p* |
| 65 | 3:1 | 27:0 | | - | - | - |  | 27:16 | 1:1 | 2.81 | ns |  | |  | 29:7 | 3:1 | 0.59 | ns |  | 23:11 | 3:1 | 0.98 | ns |  | 25:15 | 1:1 | 2.50 | ns |  | 35:2 | - | - | - |
| 3 | 5:3 | 16:11 | | 5:3 | 0.12 | ns |  | 31:12 | 5:3 | 1.69 | ns |  | |  | 32:4 | 3:1 | 3.70 | ns |  | 14:20 | 1:1 | 1.06 | ns |  | 16:26 | 1:3 | 3.84 | ns |  | 34:1 | - | - | - |
| 7 | 5:3 | 15:11 | | 5:3 | 0.26 | ns |  | 31:11 | 5:3 | 2.29 | ns |  | |  | 33:4 | 3:1 | 3.97 | ns |  | 13:18 | 1:1 | 0.81 | ns |  | 15:26 | 1:3 | 2.93 | ns |  | 34:0 | - | - | - |
| 19 | 5:3 | 18:11 | | 5:3 | 0.00 | ns |  | 33:11 | 5:3 | 2.93 | ns |  | |  | 33:4 | 3:1 | 3.97 | ns |  | 17:20 | 1:1 | 0.24 | ns |  | 16:26 | 1:3 | 3.84 | ns |  | 38:0 | - | - | - |
| 449 | 5:3 | 14:14 | | 5:3 | 1.87 | ns |  | 30:13 | 5:3 | 0.97 | ns |  | |  | 30:7 | 3:1 | 0.73 | ns |  | 13:21 | 1:1 | 3.18 | ns |  | 13:29 | 1:3 | 0.79 | ns |  | 34:2 | - | - | - |
| 453 | 1:1 | 11:15 | | 1:1 | 0.62 | ns |  | 24:17 | 1:1 | 1.20 | ns |  | |  | 29:7 | 3:1 | 0.59 | ns |  | 8:23 | 1:3 | 0.01 | ns |  | 13:27 | 1:3 | 1.20 | ns |  | 25:10 | 3:1 | 0.24 | ns |

ns= not significant, *= significant

^a^Co-1 region. Subpopulations were established using the markers CV542014 and OF10_530_; the X-Co-1 subpopulation formed by 30 RILs showing the Xana allele for these two markers, and the C-Co-1 subpopulation, formed by 47 RILs showing the Cornell49242 allele for both markers.

Co-2 region. Two subpopulations were established using the markers SQ4 and SCAReoli; X-Co-2 and C-Co-2, formed by 42 and 41 RILs showing, respectively the Xana and Cornell49242 alleles for both marker loci.

Co-3 region. Two subpopulations were established using the markers 254-G15F_550_ and SW12; X-Co-3 and C-Co-3, formed by 37 and 41 lines showing, respectively, the Xana and Cornell49242 alleles for both marker loci.
