# Supplementary material for: Genetic analysis of the response to eleven Colletotrichum lindemuthianum races in a RIL population of common bean (Phaseolus vulgaris L.)
Source: BMC Plant Biol. 2014 Apr 30;14:115. doi: 10.1186/1471-2229-14-115 (PMC4021056; doi:10.1186/1471-2229-14-115)
Supplement: Additional file 3 — Candidate resistance genes annotated from 31 to 41 Mbp of chromosome 2 and from 26 to 30 Mbp of chromosome 9, respectively, using the G19833 genotype sequence available at http://www.phytozome.net. [file 1471-2229-14-115-S3.doc]

**Additional file 3**. **In silico analyses**. Candidate resistance genes annotated from 31 to 41 M bp of chromosome 2 and from 26 to 30 M bp of chromosome 9, respectively, using the G19833 genotype sequence available at www.phytozome.net.

| Protein | Physical location  (chromosome, position in bp) | Functional annotation in the G19833 genotype | Domain^a^ |
| --- | --- | --- | --- |
| Phvul.002G232700 | Chr02: 39860936-39861934 | Serine/threonine protein kinase |  |
| Phvul.002G232600 | Chr02: 39848019-39851896 | Serine/threonine protein kinase | LRR |
| Phvul.002G232600 | Chr02: 39848019-39851896 | Serine/threonine protein kinase | LRR |
| Phvul.002G232700 | Chr02: 39860936-39861934 | Serine/threonine protein kinase |  |
| Phvul.002G237500 | Chr02: 40326916-40330333 | ATP binding | PK |
| Phvul.002G238800 | Chr02: 40493517-40495346 | Serine/threonine protein kinase |  |
| Phvul.002G238900 | Chr02: 40501069-40507895 | Serine/threonine protein kinase | LRR |
| Phvul.002G239400 | Chr02: 40558650-40561863 | Serine/threonine protein kinase | LRR |
| Phvul.002G239600 | Chr02: 40606740-40613808 | ATP binding | PK |
| Phvul.002G240100 | Chr02: 40680890-40682125 | protein binding | LRR |
| Phvul.002G242600 | Chr02: 40926783-40940156 | Serine/threonine protein kinase | LRR |
| Phvul.009G184500 | Chr09: 27179214-27183146 | Serine/threonine protein kinase | LRR |
| Phvul.009G184800 | Chr09: 27263086-27280562 | protein binding | PK |
| Phvul.009G188100 | Chr09: 27886496-27889348 | Serine/threonine protein kinase |  |
| Phvul.009G190400 | Chr09: 28205313-28209618 | Serine/threonine protein kinase | LRR |
| Phvul.009G193700 | Chr09: 28696796-28706775 | Protein binding | LRR |
| Phvul.009G195900 | Chr09: 28996271-28998838 | Serine/threonine protein kinase | LRR |
| Phvul.009G198500 | Chr09: 29363879-29365602 | serine/threonine protein kinase |  |
| Phvul.009G198800 | Chr09: 29391580-29393190 | Serine/threonine protein kinase |  |

^a^ LRR, Leucine rich repeat domain, PK, Protein Kinasa domain
